# An Essay on the Diseases of the Excreting Parts of the Lachrymal Organs

**Published:** 1819-07-01

**Authors:** 


*An Essay on the Diseases of the excreting Parts of the Lachrymal Organs.* By WILLIAM M'KENZIE, Member of the Royal College of Surgeons, and Lecturer on the Anatomy and Surgery of the Eye. Octavo. 96 Pages. London, 1819.

DURING Mr. M'Kenzie's residence on the Continent, particularly in Vienna, he paid great attention to the eye, and studied under the celebrated Beer, Professor of practical opthalmology in the University of the Austrian capital, to which Professor his present work is dedicated, in grateful remembrance of the instruction received. The contents of this little volume are in such a state of concentration already, that an analysis is impossible, without assigning to the work a very large space of our Journal. The importance of the matter may be judged of by the following table. Chap. 1. Wounds of the Lachrymal Sac. II. Erysipelatous Inflammation of the Parts covering the Lachrymal Sac. III. Acute Inflammation of the excreting Parts of the Lachrymal Organs. IV. Chronic Blenhorrhœa of the excreting parts. V. Stillicidium Lachrymarum. VI. Fistula of the Lachrymal Sac. VII. Caries of the os unguis. VIII. Relaxation of the Lachrymal Sac. IX: Mucocele of the Lachrymal Sac. X. Obstruction of the Lachrymal Canals. XI. Obstruction of Nasal Duct. We shall select a single short Chapter foi analysis, by way of exhibiting a specimen of Mr. M'Kenzie's matter and manner.

Chap. VI. *Fistula of the Lachrymal Sac.* This disease is usually the consequence of either mistreatment or neglect of the acute inflammation of the excreting parts of the lachrymal organs, or of reiterated attacks of inflammation in the same parts during the course of chronic blenorrhœa.

"If the inflamed sac be not opened at the proper time, but the collection of puriform mucus be left to itself, it will form a passage through the fibrous layer by which the sac is covered, through the orbicularis palpebrarum, and through the integuments. The opening thus formed may close soon after, and every thing go on well. But in many cases the opening contracts merely, manifests no disposition to heal, and degenerates into a fistula of the sac."

The opening in the sac and that through the integuments are rarely in opposition to each other, hence the matter forms, between the sac and the skin, several sinuses, opening by small orifices at different places. The nature of the disease is readily ascertained by pressing the finger on the superior part of the sac, when a quantity of puriform mucus will run from the fistulous opening. In patients of bad constitutions, scrofulous or syphilitic, and where the openings of the sac and integuments do not correspond, the matter often penetrates through the posterior part of the sac and os unguis, into the nose, thus causing what may be termed carious fistula. The external cicatrix is generally pretty deep, and, according to its depth and extent, it invariably produces a degree of ectropium.

"In every case of fistula, there is danger of a long continued atony of the puncta and canals, with consequent stillicidium; of disorganization of the canals from tedious suppuration or ulceration; of destruction of the sac and nasal duct from the same causes; and, in certain states of the constitution, of caries of the os unguis. If the fistula be allowed to continue for a great length of time, contraction or even obliteration of the nasal duct, from disease, is an unavoidable consequence." P. 53

The prognosis is favourable, when, on pressing the sac, a quantity of tears issues along with the morbid mucous secretion, altho' not mixed with it: — for this proves that the absorption of the tears by the puncta, and their conveyance into the sac by the canals, are restored. The restoration of the nasal duct only now remains doubtful.

"When a case of fistula of the sac presents itself, we have, first of all, to examine the fistulous opening with a fine whalebone probe, and to ascertain whether the fistulous opening of the integuments correspond or not with that of the sac. If we have convinced ourselves of the correspondence of these openings, the point of a small lancet is to be introduced into the fistula, and the opening both of the integuments and of the sac enlarged upwards and downwards. By the considerable opening thus made, a quantity of soft lint, moistened with the vinous tincture of opium, is to be passed into the sac, but not to such a depth as to fill or stop it up. Over the lint is applied a piece of adhesive plaster, and over the plaster a warm cicuta poultice with camphor. This treatment is to be continued till no trace of the fistulous hardness remains." P. 54.

When the fistula is complicated, we are to carefully ascertain the openings and the direction of the sinuses. If these last are superficial, they are to be laid open with a small bistoury, quite up to the sac. The opening into the sac is then to be enlarged upwards and downwards, as in the former case. The same treatment also, as in simple fistula, is to be adopted.

"Should one of the sinuses be so deeply seated, that, in order to lay it open it would be necessary to divide a considerable quantity of muscular substance, vessels, and nerves, we content ourselves with enlarging the fistulous opening; after which we pass a common silver probe along the sinus to its commencement in the sac, and then divide the integuments immediately over the end of the probe, so as to form a counter-opening to the sinus. Through the sinus diluted vinous tincture of opium is daily to be injected, the poultice applied as before, to promote the removal of the hardness which reigns through the sinus; and this being gained, the cure is to be completed by compression. So long, however, as hardness remains, compression is of no use;—even if the opening heal up, the sinus continues, and the opening after a while returns."

The fistula complicated with caries of the os unguis forms the snbject of another Chapter.

The whole of Mr. M'Kenzie's volume is replete with excellent matter, and forms the first of a " Series of Essays on the principal Diseases of the Eye, in which the author proposes to present to the reader an Abstract of the most valuable works which have lately appeared abroad, and especially in Germany, upon that subject, together with such observations as reflection upon what he has seen in practice may suggest." We hope Mr. M'Kenzie will perform his promise, and thus lay open the stores of continental knowledge to his insular brethren in general, and to his pupils through the medium of oral communication.

